# Characterisation of *N*-linked protein glycosylation in the bacterial pathogen *Campylobacter hepaticus*

**DOI:** 10.1038/s41598-022-26532-0

**Published:** 2023-01-05

**Authors:** Jamieson B. McDonald, Nichollas E. Scott, Greg J. Underwood, Daniel M. Andrews, Thi Thu Hao Van, Robert J. Moore

**Affiliations:** 1 School of Science, RMIT University, Bundoora West Campus, Bundoora, VIC Australia; 2 Department of Microbiology and Immunology, University of Melbourne at the Peter Doherty Institute for Infection and Immunity, Parkville, VIC Australia; 3 Bioproperties Pty Ltd, RMIT University, Bundoora West Campus, Bundoora, VIC Australia

**Keywords:** Microbiology, Molecular biology

## Abstract

*Campylobacter hepaticus* is an important pathogen which causes Spotty Liver Disease (SLD) in layer chickens. SLD results in an increase in mortality and a significant decrease in egg production and therefore is an important economic concern of the global poultry industry. The human pathogen *Campylobacter jejuni* encodes an *N*-linked glycosylation system that plays fundamental roles in host colonization and pathogenicity. While *N*-linked glycosylation has been extensively studied in *C. jejuni* and is now known to occur in a range of *Campylobacter* species, little is known about *C. hepaticus* glycosylation. In this study glycoproteomic analysis was used to confirm the functionality of the *C. hepaticus N-*glycosylation system. It was shown that *C. hepaticus* HV10^T^ modifies > 35 proteins with an *N*-linked heptasaccharide glycan. *C. hepaticus* shares highly conserved glycoproteins with *C. jejuni* that are involved in host colonisation and also possesses unique glycoproteins which may contribute to its ability to survive in challenging host environments. *C. hepaticus N-*glycosylation may function as an important virulence factor, providing an opportunity to investigate and develop a better understanding the system’s role in poultry infection.

## Introduction

*Campylobacter* is a genus of Campylobacteriacae containing species pathogenic to both humans and animals^1^. *Campylobacter jejuni* is the leading bacterial cause of foodborne gastroenteritis worldwide and together with *Campylobacter coli* cause greater than 500 million cases of enteric disease annually^2–4^. Another pathogenic *Campylobacter* species, *Campylobacter hepaticus,* has recently been identified as the cause of Spotty Liver Disease in layer hens^5,6^. SLD is a significant global problem for the poultry industry and, *C. hepaticus* can colonise the liver, small intestine, and caecum of infected birds^7^. However, unlike *C. jejuni* and *C. coli,* which are generally commensal and rarely cause serious disease in chickens, infection of layers with *C. hepaticus*, especially free-ranging and barn-raised birds, can result in the development of liver lesions, a decline in egg production of up to 25%^8^, and an increase in mortality of > 1% per day^5^. SLD has been a concern for over 20 years, predominantly in the UK and Australia^9,10^, and the incidence of disease outbreaks continues to increase across the globe, with cases also emerging in the USA^11^, Jordan^12^ and New Zealand^13^. Most disease outbreaks arise in laying hens at peak time of lay, between 22 and 35 weeks of age^14^. The global demand for egg production has tripled in the past three decades and continues to grow^15^, with increasing regulatory and consumer demands leading to a rise in barn-reared and free-range laying hen farms^14^. Consequently, without the development of effective intervention strategies the incidence of SLD is expected to continue to increase, leading to ongoing issues with egg production and mortality.

*C. jejuni* possesses an *N-*glycosylation system first discovered in 1999^16^. This is encoded by a single protein glycosylation (*pgl*) gene locus containing 10 genes which drive the modification of > 80 *C. jejuni* periplasmic, inner membrane, lipoprotein, and outer membrane surface proteins carrying a conserved heptasaccharide glycan^17–19^. While elements of the *pgl* loci are conserved across genomes of the Campylobactereriales^17^ variations in this loci, including the absence of specific glycosyltransferases or additional gene content, result in differing *N*-glycan structures^17,18^. In the closely related species *C. jejuni* and *C. coli* the *N-*glycan is a heptasaccharide containing five *N-*acetylgalactosamine residues, glucose and bacillosamine (GalNAc-α1,4-GalNAc-α1,4-[Glcβ1,3]-GalNAc-α1,4-GalNAc-α1,4-GalNAc-α1,3-diNAcBac-β1 (where diNacBac is di-N-acetlybacillosamine2,4-diacetamido-2,4,6 trideoxy-D-glucopyranose)^20^. The heptasaccharide is synthesized on the lipid carrier undecapprenyl pyrophosphate (Und-P) linked to diNAcBac, arranged and formed by PglF, PglE and PglD. The repeating Gal*N*A*c* sugar chain is then constructed by glycosyltransferases PglA*,* PglC*,* PglH*,* and PglJ with the addition of glucose by PglI. The synthesised heptasaccharide is exported across the inner membrane into the periplasm by the flippase PglK. Central to the *N-*glycosylation locus is *pglB*, which encodes an oligosaccharyltransferase and is responsible for the transfer of the *N-*glycan heptasaccharide structure to an asparagine residue, within a conserved D/E-X-N-X-S/T sequon in acceptor proteins in the periplasm^19,20^. This *N*-glycosylation system has been shown to play crucial roles in epithelial cell adherence, invasion, and colonisation in chickens^21–23^, as well as in human infection and pathogenicity^24^. *Campylobacter* devoid of this function are unable to colonize the natural chicken host^25^, further highlighting the importance of the system in promoting survival.

The similarities and differences in the protein *N-*glycosylation pathways within the *Campylobacter* genus have been characterised using glycopeptide enrichment coupled with high resolution tandem mass spectrometry. This technique has proven to be valuable in understanding *Campylobacter* glycoproteomes such as *C. jejuni* and its role in pathogenicity^17–19,26^. Indeed, recent work using glycopeptide analysis has demonstrated that even within *C. jejuni,* differences in the *N*-linked glycan do exist at a population level^27^. In contrast to the well-studied *C. jejuni*, understanding of *C. hepaticus* pathogenicity is in its infancy owing to its recent discovery^6^. Therefore, this study aimed to confirm *N*-glycosylation in *C. hepaticus* and assess the similarity of the *N*-glycan(s) used in *C. hepaticus* to that of the C. *jejuni* heptasaccharide*.* Furthermore, the study also aimed to identify glycoproteins of *C. hepaticus* as potential virulence factors possibly linked to colonisation and survival in the chicken host. Glycopeptide enrichment and non-enrichment strategies coupled with high resolution tandem mass spectrometry were used to provide the first evidence that *C. hepaticus* glycosylates proteins in a manner most closely related to that seen in *C. jejuni* and *C. coli*.

## Results

**Figure 1:**
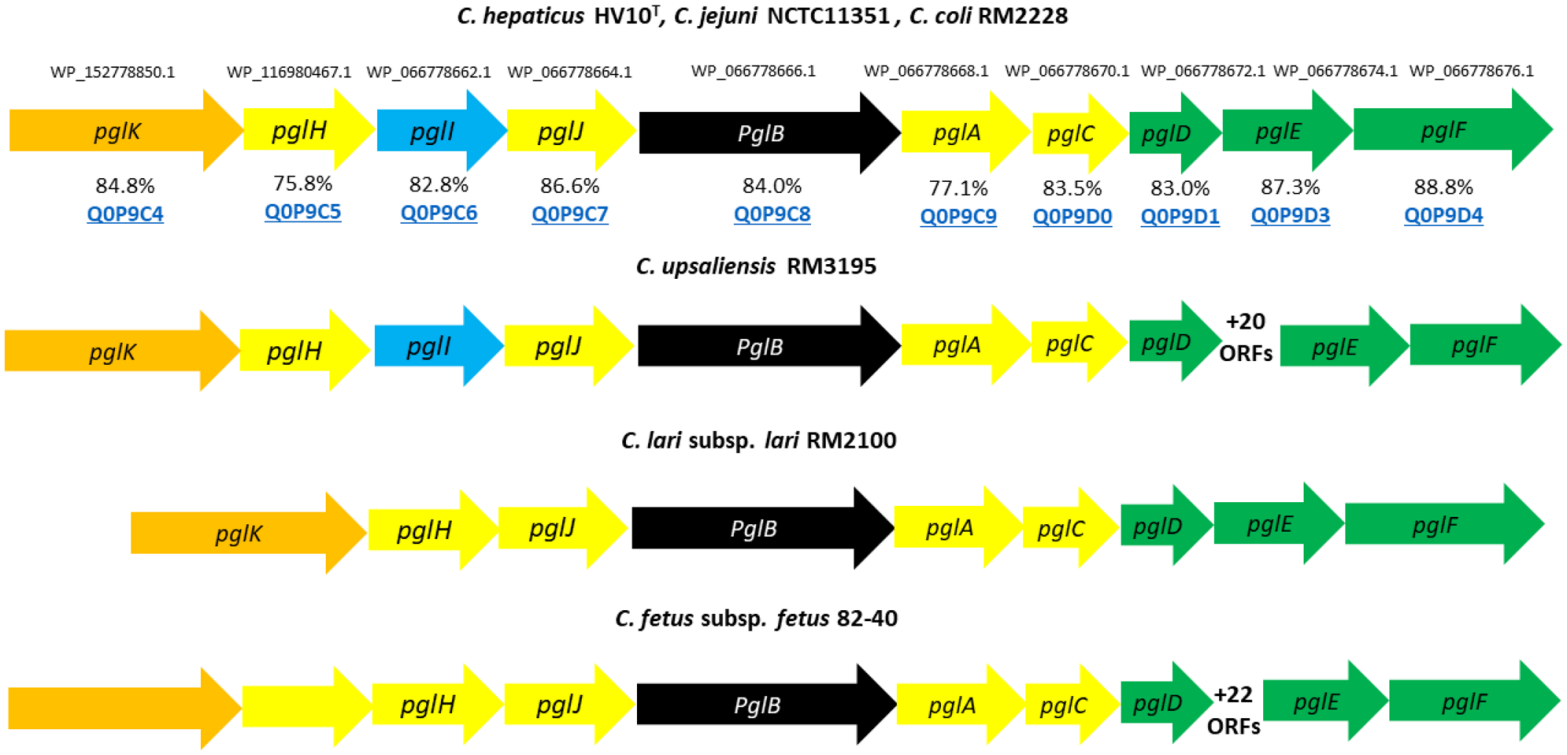
Comparison of *pgl* loci organisation in *Campylobacter* species. *C. hepaticus* HV10^T^, *C. jejuni* NCTC 11351 and *C. coli* RM2228 share the same arrangement of genes in the *pgl* locus. The coding sequence accessions for each *C. hepaticus* HV10^T^ *pgl* gene are included above each gene and the individual gene products/protein identity (%) compared to *C. jejuni* subsp. *jejuni* NCTC11168 encoded gene products are stated below with the Uniprot entry accession number. The genetic organisation of this *pgl* locus has been compared to other more distantly related species (according to^17^). Genes encoding glycosyltransferases are shown in yellow, *pglB* in black, genes involved in diNAcBAc synthesis in green, and flippase in orange.

To determine the *N*-linked glycosylation potential of *C. hepaticus* the *pgl* locus within the *C. hepaticus* HV10^T^ genome was annotated and investigated. *C. hepaticus* HV10^T^ encodes all the genes required for the generation of a heptasaccharide *N-*glycan. Orthologs of the C. *jejuni* Und-PP synthesis genes (*pglD, pglE and pglF*), glycosyltransferase genes (*pglA, pglC, pglH, pglI and pglJ*), the flippase gene (*pglK)*, and the oligosaccharyltransferase, *pglB* gene, were identified. The arrangement of these genes in *C. hepaticus* HV10^T^ is highly conserved with that of *C. coli* RM2228 and *C. jejuni* NCTC11351 (Fig. 1). A Uniprot BlastX of each gene’s nucleotide sequence within the *C. hepaticus pgl* locus revealed varying levels of protein sequence identity compared to *C. jejuni subsp. jejuni* NCTC 11168 (Fig. 1), indicating some degree of variation between the species. It should be noted that *C. coli* RM2228 and *C. hepaticus* HV10^T^ lack the Cj1122c gene between the *pglD* and *pglE* in the *C. jejuni subsp. jejuni* NCTC 11168 locus. However, the presence of this gene is uncommon across other *C. jejuni* strains. In addition, the function of this gene’s product remains unknown but does not function as a part of *N-*glycan biosynthesis^28^.

The arrangement of the *pgl* genes in more distantly related *C. lari* subsp. *lari* RM2100 and *C. fetus* subsp. *fetus* 82–40 varies with that of *C. hepaticus* HV10^T^, *C. jejuni* NCTC 11351, and *C. coli* RM2228, impacting the overall *N-*glycan structure. For example, *C. lari* subsp. *lari* RM2100 and *C. fetus* subsp. *fetus* 82–40 both lack *pglI,* and therefore glucose is absent from these species *N-*glycan. Comparison of *C. hepaticus* HV10^T^ and *C. fetus* subsp. *fetus* 82–40 indicates a further division of these species, with *C. fetus* subsp. *fetus* possessing extra ORFs encoding putative flippases and glycosyltransferases.

**Figure 2:**
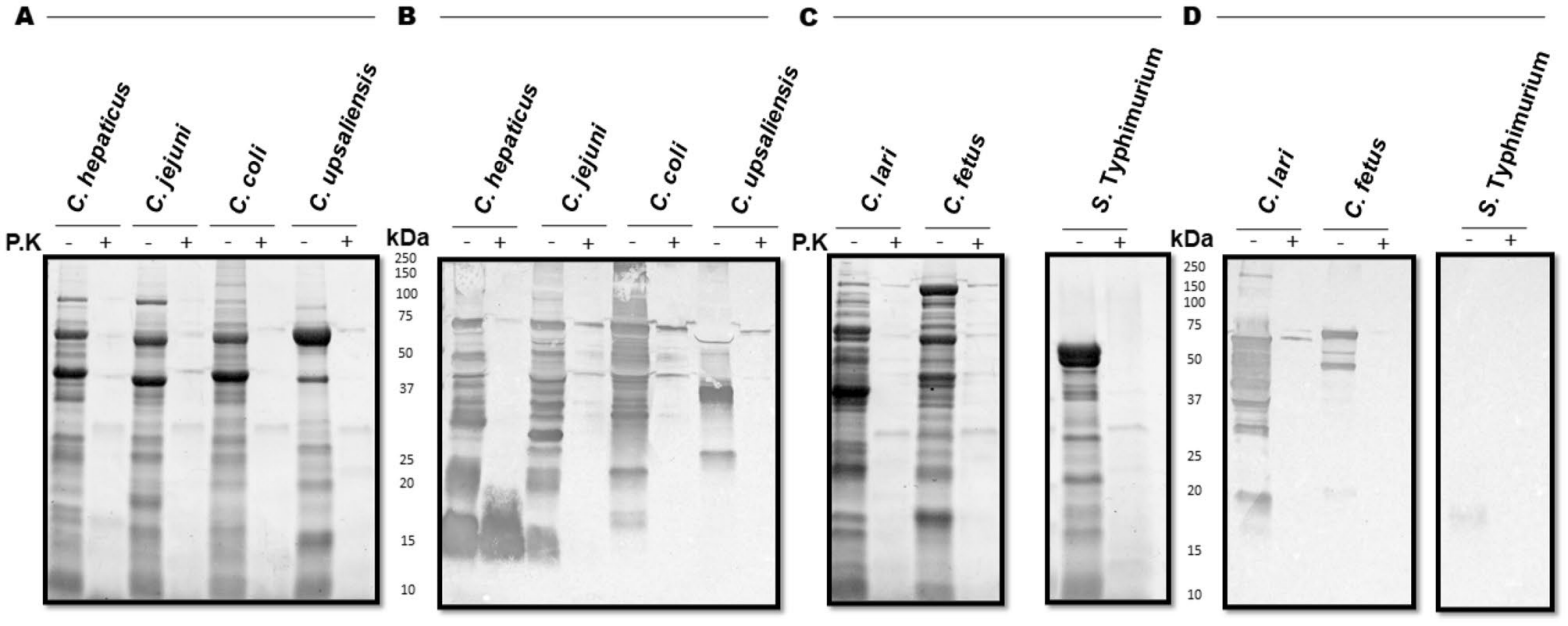
(**A**) SDS-PAGE of *C. hepaticus* HV10^T^, *C. jejuni* 354, *C. coli* 52/2 and *C. upsaliensis* 54/7 whole cell lysates (WCL) containing 25 µg of protein. (**B**) SBA lectin blot binding profiles to *N-*glycans present in WCL of *C. hepaticus* HV10^T^, *C. jejuni* 354, *C. coli* 52/2, *C. upsaliensis* 54/7 containing 25 µg of protein. (**C**) SDS-PAGE of *C. lari* 54/6, *C. fetus* 54/3 alongside negative control, *S.* Typhimurium PT44. The equivalent amount of protein from WCL of the different *Campylobacter* species were also digested with proteinase K (P.K). Whole cell lysates were separated by 8–16% SDS-PAGE and either developed with SimplyBlue™ SafeStain or transferred to PVDF membranes for blotting. (**D**) SBA lectin blot binding profiles to *N-*glycans present in WCL of *C. lari* 54/6, *C. fetus* 54/3 and *S.* Typhimurium PT44 containing 25 µg of protein.

To experimentally determine whether *C. hepaticus* HV10^T^ modifies proteins with an *N-*glycan composed of terminal α- or β- linked *N*-acetylgalactosamine residues, the binding of soybean agglutinin lectin (SBA), known to interact with the terminal α- or β linked GalNac residues of the *C. jejuni N*-glycan heptasaccharide^29^, to *C. hepaticus* HV10^T^ soluble proteins, was investigated (Fig. 2). SBA bound to various *C. hepaticus* HV10^T^ proteins. Binding patterns to *C. hepaticus* HV10^T^ proteins shared similarities with *C. jejuni* 354 and *C. coli* 52/2, likely indicating the presence of homologous *N-*glycoproteins. A few signals were unique to *C. hepaticus* HV10^T^suggesting some differences in the glycosylated proteins between these species. *C. hepaticus* forms a separate clade from other known *Campylobacter* species, which is positioned close to *C. jejuni* and *C. coli*^30^. This would modestly explain the similarities in SBA binding patterns between these species and the differences seen in comparison to *C. upsaliensis*.

The profile of proteins that SBA bound to differed considerably from more distantly related species. SBA binding was reduced considerably in *Campylobacter fetus* subsp. *fetus*, which modifies proteins with a different *N-*glycan structure that lacks a terminal GalNAc residue; thus, SBA does not preferentially bind^17,18^. The SBA reactive bands present in the *C. fetus* subsp. *fetus* have previously been attributed to non-specific binding^18^. Loss of protein bands in the SDS-PAGE loading control and SBA binding signals in the lectin blots of proteinase K digested samples supports the assumption that bands in the lectin blots corresponded to proteins with glycan modifications despite the presence of some weak upper bands, which is likely due to incomplete digestion of protein.

Interestingly, within *C. hepaticus* HV10^T^, a dominant band at approximately 15 kDa was found, which was absent in all other *Campylobacter* profiles, this band was determined to be non-proteinaceous as it was present in the equivalent proteinase K digested sample. It is hypothesized that this band indicates the addition of a glycan containing α- or β- linked GalNac as a part of the lipooligosaccharide (LOS).

The relative signal strengths against *C. hepaticus* HV10^T^ *N-*glycoproteins appear to be slightly weaker than binding to *C. jejuni* 354 and *C. coli* 54/2 proteins, with most of the signal strength corresponding to the non-proteinaceous 15 kDa band. A faint band present in *S.* Typhimurium PT44 may correspond to SBA binding non-specifically to galactose residues^31,32^. But the specificity of the SBA binding indicating the presence of the *N*-linked glycan was confirmed by the absence of significant signals in *S.* Typhimurium PT44 (Fig. 2 and Supplementary Fig. 1), which lacks the *pgl* locus required for glycosylation.

**Figure 3:**
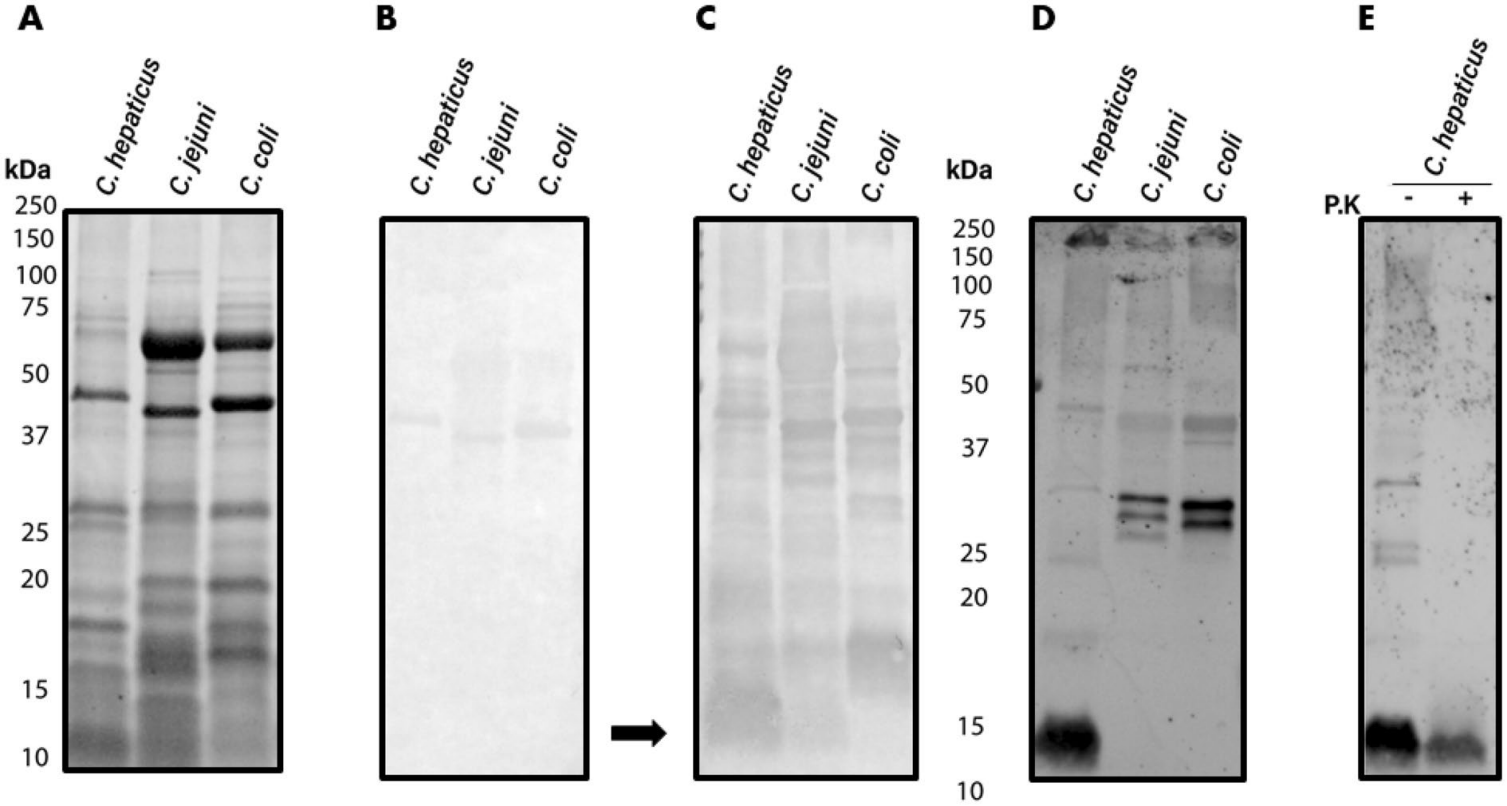
Binding of serum IgY antibodies from *C. hepaticus* HV10^T^ bacterin vaccinated birds to *Campylobacter* whole cell lysates. Wells were loaded with whole cell lysates containing 15 µg of protein, (**A**) SDS-PAGE loading control, (**B**) immunoblot with *C. hepaticus* negative sera (1:200) (**C**) Immunoblot of *C. hepaticus* HV10^T^ positive (3 times vaccinated with *C. hepaticus* HV10^T^ bacterin) sera (1:200). The arrow points to an immunodominant band corresponding to the ≈ 15 kDa non-proteinaceous signal suspected to be *C. hepaticus* HV10^T^ LOS (**D**) SBA lectin blot (**E**) SBA lectin blot after proteinase K digestion (P.K).

To investigate the potential immunoreactivity of *C. hepaticus N*-glycoproteins identified by lectin blotting we examined whole cell lysates probed with sera from birds vaccinated with *C. hepaticus* HV10^T^ bacterin to identify any potential immunodominant bands. Immunoblotting revealed a strong IgY binding profile to whole cell lysates of *C. hepaticus* HV10^T^*, C. jejuni* 354 and *C. coli* 54/2. IgY binding to *C. jejuni* 354 and *C. coli* 54/2 (Fig. 3 and Supplementary Fig. 2) was expected due to the presence of homologous proteins and most mature birds will have also been exposed to these bacteria^33^. The strong binding profiles made it difficult to decipher immunoreactive signals corresponding to specific *N-*glycoproteins seen in the lectin blot. Despite this, there was an immunoreactive band corresponding to the 15 kDa non-proteinaceous signal present in *C. hepaticus* HV10^T^ while this band was faint (possibly corresponding to the 15 kDa *C. jejuni N-*glycan seen in Fig. 2) in *C. jejuni* 354 or absent in *C. coli* 54/2.

To further confirm the presence of the *N-*glycosylation glycoproteomic analysis using ZIC-HILIC^26^ and FAIMS^34^ based methods were undertaken on *C. hepaticus* HV10^T^ with the resulting data analysed using open database searching^35,36^. ZIC-HILIC is a standard method used to enrich glycopeptides by hydrophilic partitioning. In contrast, FAIMS can be applied to total protein samples (enrichment-independent) and provides an alternative means to identify glycopeptides. ZIC-HILIC typically requires large amounts of starting material, whereas FAIMS has been shown to be particularly useful when starting with low input amounts^34^. Thus, in combination, these techniques could improve glycopeptide coverage.

**Figure 4:**
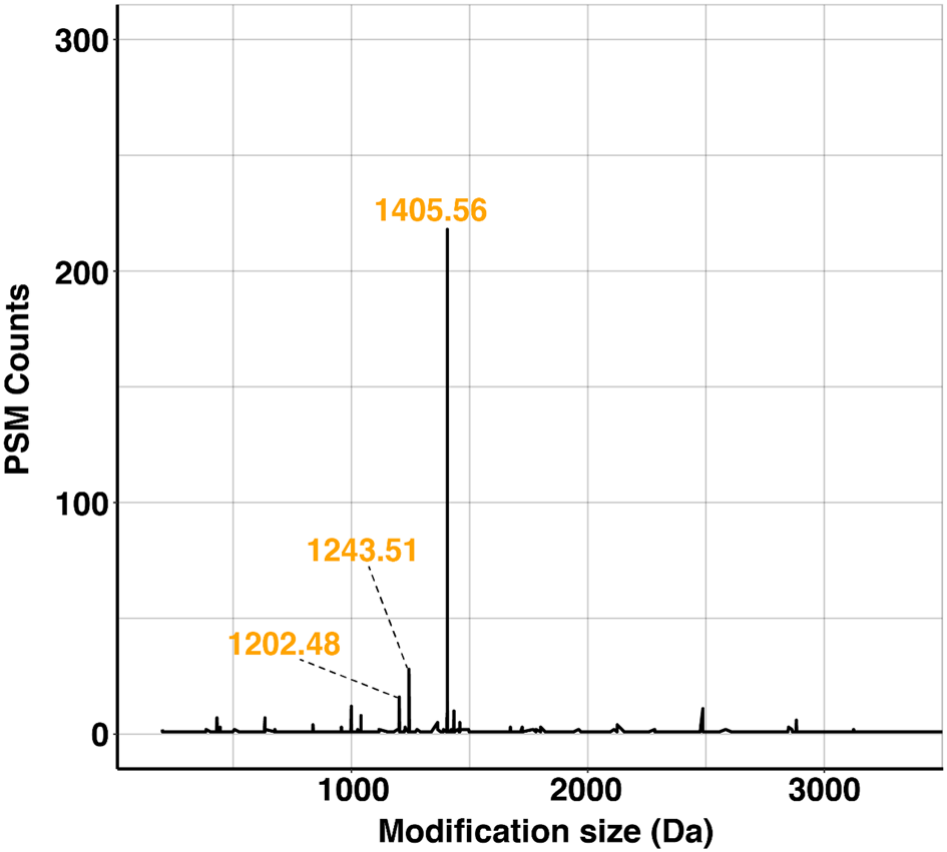
Open searching results (data files searched using MSfragger (v15)) of *C. hepaticus* HV10^T^. Open database searching revealed the presence of a 1405.56 Da modification, consistent with the mass expected of *C. jejuni* heptasaccharide glycan. In addition, masses of 1243.51 and 1202.48 Da, corresponding to the loss of Hex and HexNAc from the heptasaccharide, were detected.

Examination of the potentially modified peptides observed with this approach revealed a dominant modification mass of 1405.56 Da (Fig. 4, Supplementary Table 1), consistent with the expected mass of *C. jejuni* glycan (1405.560 Da), in addition to masses corresponding to hexasaccharides of 1243.51 and 1202.48 Da resulting from the loss of Hex and HexNAc from the dominant heptasaccharide. Open searching also revealed additional glycoforms such as formylated glycan corresponding to a mass of 1433.56 (1405.56 Da + 27.99 Da, Supplementary Fig. 3 and Supplementary Table 1). As formylation of glycans has previously been detected^17,26,35^ and shown to be undesirable modification of glycopeptides, due to the use of high concentrations of formic acid or storage of samples in formic acid during sample preparation and enrichments steps, these are unlikely to be biologically relevant.

**Figure 5:**
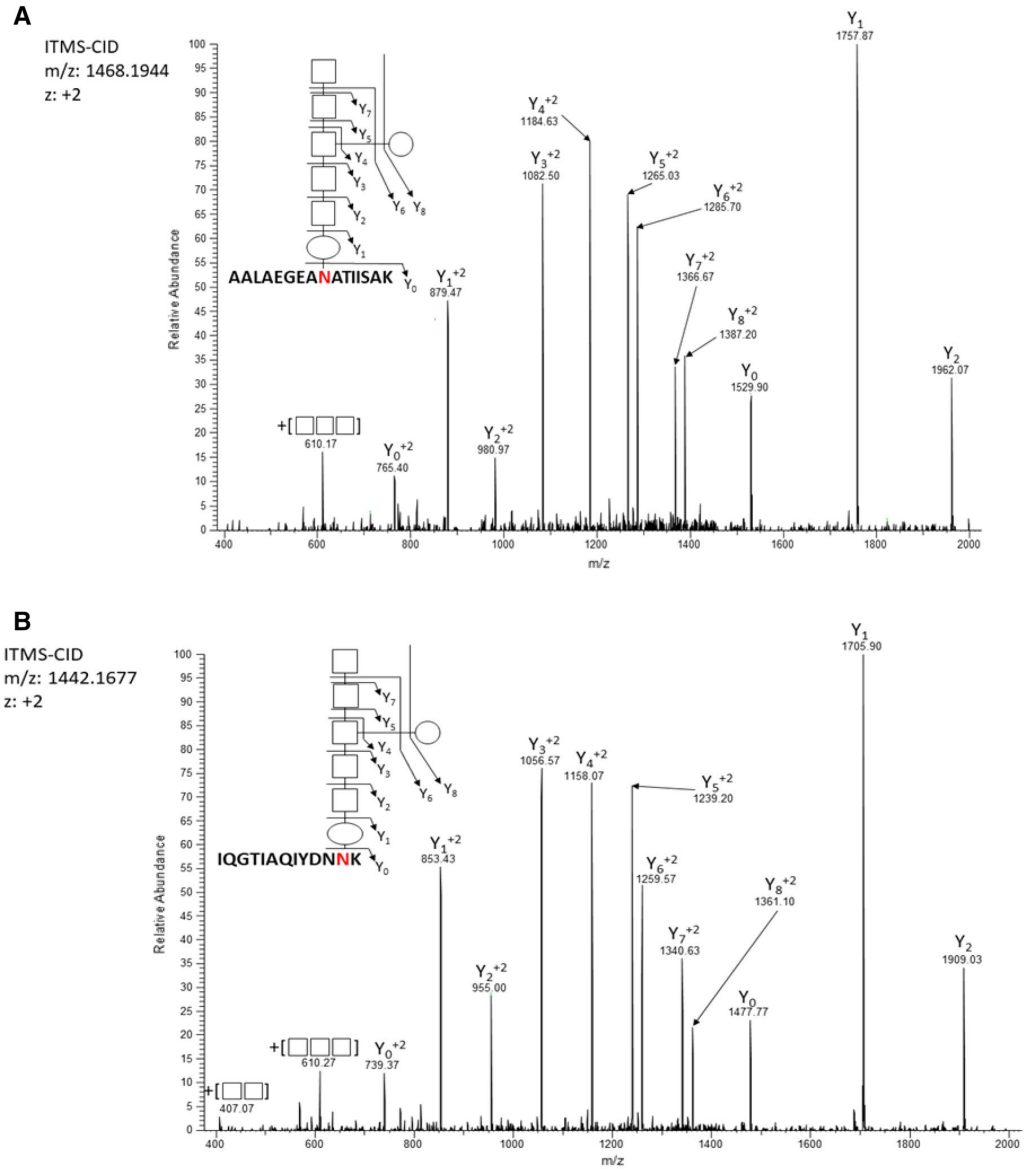
Ion-trap CID-MS spectra of two *C. hepaticus* HV10^T^ glycopeptides. Spectra represent fragmented ions produced from the *N-*glycan. (**A**) *C. hepaticus* HV10^T^ glycopeptide ^266^AALAEGEANATIISAK^282^ with a delta mass of 1405.56 Da. (**B**) *C. hepaticus* HV10^T^ glycopeptide ^23^IQGTIAQIYDNNK^36^ with a delta mass of 1405.56 Da. Above is the heptasaccharide structure linked to the peptide drawn using the symbol nomenclature for glycans SNFG. The MS/MS spectra display fragmented ions generated from the glycan with the structure HexNAc-HexHAc-[Hex]-HexNAc-HexHAc-HexNAc-diNAcBac.

To further support the identity of the 1405.56 Da modification of *C. hepaticus* as a heptasaccharide *N*-glycan, ion trap-based CID scans of peptide decorated with the 1405.56 Da modification were further assessed. Manual annotation of Ion-trap CID spectra from two such glycopeptides (Fig. 5) demonstrated that the 1405.56 Da modification is consistent with a glycan of HexNAc-HexHAc-[Hex]-HexNAc-HexHAc-HexNAc-diNAcBac which agrees with the well characterised *C. jejuni* heptasaccharide glycan, GalNAc-GalNAc-[Glc]-GalNAc-GalNAc-GalNAc-diNAcBac. Together these analytical methods confirmed that *C. hepaticus N*-linked glycosylation is consistent with the previously identified *C. jejuni* structure and, in combination with the SBA lectin blot data strongly suggests that *C. hepaticus* modifies peptides with the same *N-*glycan heptasaccharide as its two most closely related *Campylobacter* species, *C. jejuni* and *C. coli*.

**Figure 6:**
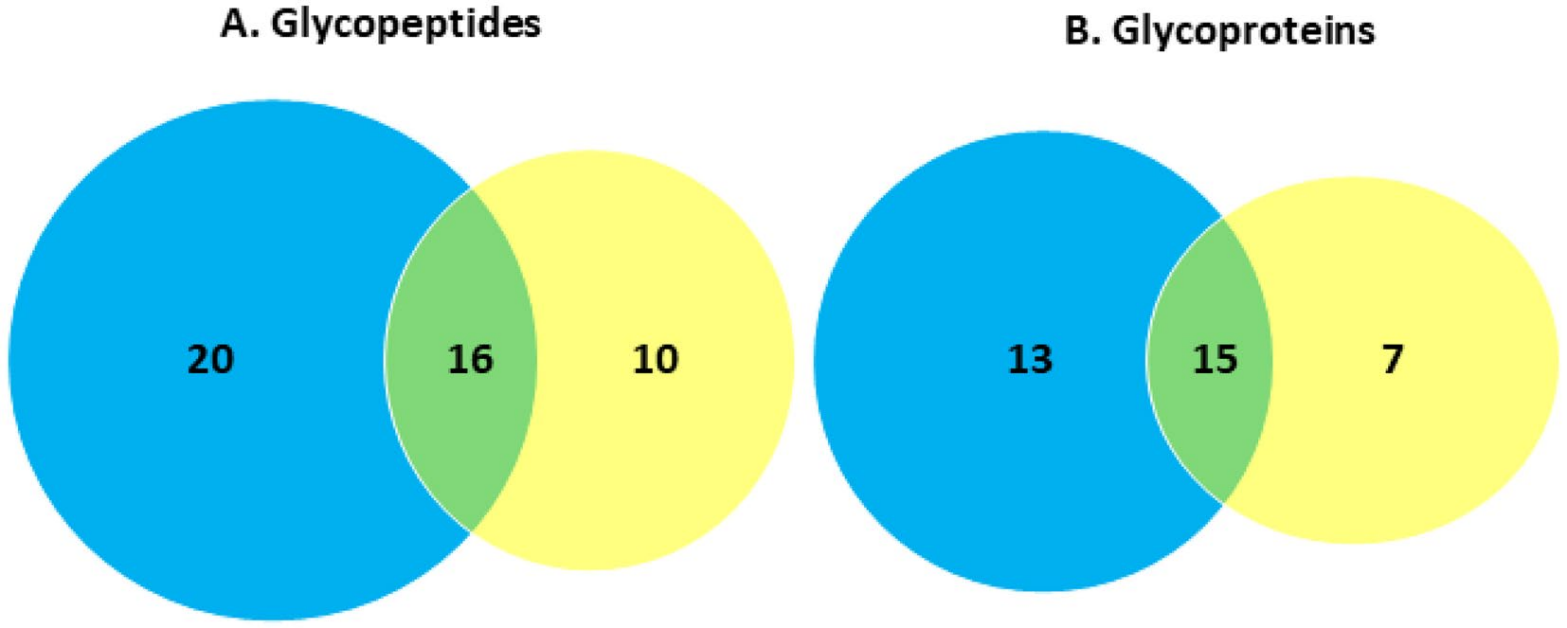
Overlap of *C. hepaticus* glycopeptides (**A**) and glycoproteins (**B**) identified using FAIMS (blue) and ZIC-HILIC (yellow) enriched samples. Combined, 46 glycopeptides from 35 glycoproteins were identified.

To provide a diverse cross section of the *C. hepaticus* glycoproteome two glycopeptide enrichment strategies were employed, ZIC-HILIC and FAIMS enrichments. While ZIC-HILIC glycopeptide enrichment enabled the Identification of 26 glycopeptides, corresponding to 22 glycoproteins, FAIMS enabled the identification of 36 glycopeptides corresponding to 28 glycoproteins (Fig. 6). While many of the glycopeptides and proteins identified were shared between the two approaches the majority were unique to a single enrichment approach, resulting in the identification of 46 glycopeptides corresponding to 35 glycoproteins (Fig. 6).

The identity, function, species diversity, localization, and abundance of the proteins identified as glycosylated were assessed using Uniprot peptide search, BLAST, Gneg-mPloc, PSORTb (vers. 3.0.3.), and iBAQ analysis, respectively (Supplementary Fig. 4 Supplementary Table 2).

All peptides confirmed to be glycosylated possess the conserved consensus sequon D/E-X-N-X-S/T, the same as the sequon identified in *C. jejuni*^19^. The putative localization of the glycoproteins included the outer membrane (4) and periplasm (2), with the majority (23) of the proteins predicted to be localised in the inner membrane. A single flagellar protein, homologous with *C. jejuni* PflA, localized in the cytoplasm (Supplementary Table 2). Although *N-*glycan modification occurs exclusively to non-cytoplasmic proteins it’s important to consider the topology of such proteins^19^. In C. *jejuni* the PflA protein product is positioned in the periplasm and therefore is capable of being glycosylated^19^ and results suggest this to be the case for the *C. hepaticus* HV10^T^ equivalent. However, this could not be determined using topology prediction software.

Glycosylation of some proteins was identified to be unique to *C. hepaticus* HV10^T^ (Table 1 and Supplementary Table 2) or modification of these proteins, if homologs exist, have not yet been experimentally identified in *C. jejuni*. These glycoproteins included a fibronectin type III domain-containing protein, a MetQ/NlpA family ABC transporter substrate-binding protein, a SH3 domain containing protein, and an ArsS family sensor histidine kinase. A PDZ domain-containing protein, identified as glycosylated in *C. hepaticus* HV10^T^*,* contained an identical sequon in the *C. jejuni* subsp. *jejuni* NCTC11168 homolog however glycosylation of the *C. jejuni* protein is yet to be experimentally confirmed. Many of the other glycoproteins share > 80% sequence identity with homologous *C. jejuni* glycoproteins that have been experimentally verified. The remaining protein targets identified were uncharacterized/hypothetical proteins which showed some degree of sequence identity (< 80%) with identified *C. jejuni* subsp. *jejuni* NCTC11168 glycoproteins. Proteins were predicted to be found in the inner membrane, outer membrane, or periplasm, while for some the subcellular localization couldn’t be predicted. Nine out of thirty-three identified glycoproteins were modified at two or more sites. For example, the OmpA family protein, and AMIN domain protein contain three canonical sequons (Supplementary Table 2) and share 89.6%, and 84% sequence identity, respectively with known *C. jejuni* subsp. *jejuni* NCTC11168 glycoproteins. For some glycosylated proteins the number of sequons differed between *C. hepaticus* HV10^T^ and *C. jejuni* subsp. *jejuni* NCTC11168. For example, two sequons have been identified in the *C. jejuni* subsp. *jejuni* NCTC11168 periplasmic fusion protein CmeA (multi-drug antibiotic efflux system CmeABC protein), whereas only a single sequon was identified in the *C. hepaticus* HV10^T^ homolog (81.2% identity). Conversely, a single sequon has been identified in the *C. jejuni* subsp. *jejuni* NCTC11168 multidrug efflux transporter periplasmic adapter subunit CmeE, whereas two sequons were identified in the *C. hepaticus* HV10^T^ homolog (76.8%). This was not surprising as glycosylation sites have been shown to vary amongst *C. jejuni* strains^37^.

**Table 1:** *N-glycoprotein* determinants of *C. hepaticus* possibly involved in niche adaption and virulence.

Glycosylation status	Protein name (UniProt accession)	Similarity to *C. jejuni* (UP000411585) proteins^#^	Glycosylation sites	Peptide sequence/*N*-glycan sites	Localisation	Regulation *in* bile isolated from SLD positive birds
Shared with *C. jejuni*	**A0A424Z313** Efflux RND transporter periplasmic adaptor subunit	81.2% identity **Q0PBE3** Periplasmic fusion protein CmeA	1	^98^ANVDIAYGQTLMAQANFENASKDF**N**RS^124^	Inner membrane	No change
	**A0A6A7JTI1** OmpA family protein	89.6% identity **Q0PAR8** OmpA family protein	3	^166^LDD**N**ITVDEK^176^ ^108^DL**N**STLDDKDKQ^119^ ^93^AELEA**N**ITNYKQ^104^	Outer membrane	Unknown
	**A0A424Z2F4** Nitrate reductase, electron transfer subunit	82.7% identity **Q0PAA7** Nitrate reductase cytochrome c-type subunit	1	^40^LVEA**N**FTSLQPGESTRL^56^	Periplasm	Upregulated
Unique or not yet identified in *C. jejuni*	**A0A6A7JR14** MetQ/NlpA family ABC transporter substrate-binding protein	85.6% identity **Q0PAB8** Putative NLPA family lipoprotein	1	^54^EFTDYVLPNLAVDNAEIDANFFQHTPYLEEF**N**KS^87^	Inner membrane	Unknown
	**A0A424Z0D4** SH3 domain containing protein	72.8% identity **Q0P9V3** Putative lipoprotein	1	^39^LEFEQ**N**VSILPKL^51^	Inner membrane	Unknown
	**A0A6A7JQY3** ArsS family sensor histidine kinase/HAMP domain containing family sensor histidine kinase	78.2% identity **Q0P8Z4** Two component sensor	1	^48^NLSLFYEN**N**ISNAKI^62^	Inner membrane	Unknown
	**A0A424Z1P2** PDZ domain-containing protein	83.5% identity **Q0P7Y5** putative periplasmic protein containing protein	1	^233^DNQDL**N**ISTEIFAKD^247^	Inner membrane	Unknown
	**A0A6A7JR07** Fibronectin type III domain-contaiing protein/ Ferrous iron transporter A	80.5% identity **Q0P8X7** Putative fibronectin domain containing protein	1	^126^IIEA**N**TTSRL^35^	Inner membrane	Unknown
Unknown	**A0A424Z2X7** Hypothetical protein	84.3% identity—**Q0PBE6** hypothetical protein	1	^18^EQFYSYFDQ**N**ISK^30^	Inner membrane	Unknown

^#^Protein identity was determined by NCBI BLAST and Uniprot peptide search; no. of glycosylation sites for each protein; the peptide sequences and *N*-glycan site (manually assigned) based on consensus sequon **D/E-X-N-X-S/T**. Localisation was determined using Gneg-mPloc & PSORTb (vers. 3.0.3). In vivo gene regulation of glycoproteins identified were crossed referenced with the findings of Van et al.^7^.

Potential in vivo regulation of glycoprotein expression was investigated by cross referencing the glycosylated protein list with the gene expression findings of Van et al.^7^ to determine if genes associated with colonization of the bile were also glycosylated (Table 1). Nitrate reductase was found to be upregulated in *C. hepaticus* recovered from the gallbladder of SLD positive birds (Table 1). Other glycoproteins such as efflux RND transporter and the TolC family protein were reported to have no change in gene expression. The remaining glycoproteins either showed no change or change in gene regulation wasn’t reported.

## Discussion

The *Campylobacter N*-glycosylation pathway is understood to play a critical role in host colonization, invasion, and pathogenicity^21–23^. Because *C. jejuni* and *C. hepaticus* are closely taxonomically related, it was hypothesized that *C. hepaticus* HV10^T^ would also possess this system. This study provides strong evidence indicating that *C. hepaticus* HV10^T^ glycosylates proteins with an *N-*linked heptasaccharide glycan composed of terminal GalNac residues like the well-characterized *C. jejuni N-*glycan. The identified *C. hepaticus N-*glycoproteins are modified at the same sequon as closely related species *C. jejuni* and *C. coli*. These are protein homologs associated with host colonization and survival in *C. jejuni*. In addition, *C. hepaticus* possesses unique glycoproteins that may contribute to its ability to traffic from the gastrointestinal tract to physiologically challenging sites of infection, such as the gall bladder, where it can survive and induce disease in the liver.

Analysis of whole genome sequence data and lectin blotting with SBA^29^ provided preliminary evidence that *C. hepaticus* HV10^T^ has the genes required for *N-*glycosylation and does indeed modify proteins with an *N-*glycan consisting of α- or β-linked GalNAc residues. A dominant signal corresponding to a 15 kDa band suspected to be a non-proteinaceous glycoconjugate was also seen in *C. hepaticus* HV10^T^ but absent in all other campylobacters screened. Immunoblotting showed this band to be highly immunogenic in *C. hepaticus* HV10^T^ vaccinated birds. It is hypothesised that this band corresponds to LOS. LOS is highly variable in structure among campylobacters, with the chromosomal regions encoding its synthesis often changed by rearrangements and recombination events^38^. It is also possible that *C. hepaticus* HV10^T^ LOS contains terminal GalNac residues as part of their glycan constituent to which SBA can bind. Genome sequencing has revealed *C. hepaticus* HV10^T^ to have a 6.6 kb insertion of seven predicted coding sequences into the *cst-II* gene between *waaF* and *waaC* genes of the LOS locus, all of which are predicted to function as glycosyltransferases^7^. Approximately one third (2 kb) of the inserted sequence is exclusive to *C. hepaticus* HV10^T^, with the remaining 4.6 kb displaying high sequence divergence to *C. jejuni* isolates^7^. A better understanding of these unique coding sequences may provide insights into their products’ roles in niche adaptation and virulence.

Several glycoproteins associated with motility, chemosensing, and chemotaxis, as well as adhesins including fibronectin binding proteins are essential in *C. jejuni* colonisation^39^. This study identified glycoproteins involved in *Campylobacter* host colonisation common to both *C. jejuni* subsp. *jejuni* NCTC11168 and *C. hepaticus* HV10^T^. For example, the putative family OmpA protein (chemotaxis protein MotB) is an important component of the stator complex of flagellar, and for providing energy for rotation and flagellar function^40,41^. Loss of flagellar motility and mutagenesis of serine protease CtpA in C. *jejuni* severely hinders colonisation in chickens^42^.

Other glycoproteins identified had some degree of sequence similarity with characterized proteins from *C. jejuni* subsp. *jejuni* NCTC11168 and other organisms, but their functions regarding colonization, cell adherence, and virulence remain largely unknown. However, glycosite mutations in *C. jejuni cmeA*, which encodes an antibiotic efflux glycoprotein also identified in the current study as glycosylated in *C. hepaticus* HV10^T^, have shown that *N*-glycosylation is required for optimal function^25,43^. CmeA mutants have enhanced susceptibility to multiple antimicrobials such as bile salts and ciprofloxacin, and importantly have a reduced capacity to colonise chickens^44^. Evidence has also demonstrated vital roles in cell binding through glycan-glycan interactions and protein stability by providing protection against host proteolytic functions^25^. Due to the high conservation of these proteins, they likely play similar interspecies roles in survival and pathogenicity.

C. *hepaticus* has genetically diverged from other *Campylobacter* species and this is correlated with its unique ability, amongst campylobacters, to efficiently colonise and persist within the bile and cause extensive liver disease in chickens. It is clear C. *hepaticus* HV10^T^ possesses several homologous glycoproteins associated with colonization and infection of *C. jejuni* in chickens. However, an invasive assay using chicken liver cells revealed *C. hepaticus* to be more invasive than other *Campylobacter* species, including *C. jejuni*^5^. Therefore, *C. hepaticus* is likely to have more effective or additional mechanisms associated with cell invasion and colonization that are not found in *C. jejuni* or *C. coli*. Interestingly, glycosylation of an ArsS family sensor histidine kinase is unique to *C. hepaticus.* This enzyme is of particular interest as it is not found in *C. jejuni,* although it plays an important role in the regulation of acid adaptation in *Helicobacter pylori*^45,46^. ArsS regulates acid-induced membrane trafficking of urease and its additional proteins to the inner membrane, facilitating rapid, urea-dependent cytoplasmic and periplasmic buffering, aiding survival in acidic conditions^6^. Similarly, protein *N-*glycosylation was demonstrated to be critical for nitrate reductase (NapA/NapB) function in *C. jejuni* where organisms lacking the glycosylation machinery had significantly reduced levels of all the nitrate reductase respiratory proteins which are required under low oxygen conditions^7,25^. Further, a NapA mutant of *C. jejuni* had a significantly reduced ability to colonise the ceca of chickens when compared to the wild type, indicating *N*-glycosylation is required for a Nap nitrate reductase enzyme activity^25,47^. In *C. hepaticus* HV10^T^, a component of the periplasmic nitrate reductase NapAB complex, nitrate reductase electron transfer subunit (NapB), was identified as glycosylated at a single site with upregulation of this gene in *C. hepaticus* recovered from the bile of SLD positive chickens^7^, further emphasizing the importance of the ability of *C. hepaticus* to establish infection in the oxygen-deprived environment found in the bile. Thus, further investigation of these glycoproteins is warranted as they may play important roles in the ability of *C. hepaticus* to traffic through the acidic environment of the GI tract and colonize in the challenging environment of the bile.

It is suspected that there are additional *N*-glycan targets to be identified within the *C. hepaticus* glycoproteome. Scaling up the growth, harvest, and extraction of proteins from *C. hepaticus* may allow the identification of lower abundance proteins that are glycosylated. Furthermore, future studies may consider performing a multiple-protease digestion on samples by utilizing enzymes alternative to trypsin which could expand sequon coverage and glycopeptide identification. This in turn may result in the identification of more unique glycoproteins associated with the ability of *C. hepaticus* to proliferate in the bile and induce disease in the liver of layer hens.

In summary, this study confirmed that *C. hepaticus* encodes *N-*glycosylation machinery, which modifies proteins with an *N-*linked heptasaccharide glycan composed of terminal GalNac residues closely comparable to the *N-*glycanfound in *C. jejuni* and *C. coli*. Future studies utilizing nuclear magnetic resonance are required to provide a complete structural characterization of *C. hepaticus* HV10^T^ *N-*glycan to unequivocally determine whether this *N-*glycan has the same structure as *C. jejuni*. Nevertheless, twenty-nine glycoprotein homologs were identified to be shared with *C. jejuni*. Five other glycoproteins, where glycosylation is unique to *C. hepaticus* HV10^T^ or has not yet been identified in other *C. jejuni* were identified*.* It is hypothesized that the *N-*glycan heptasaccharide modification of *C. hepaticus* proteins may play a role in niche survival mechanisms involved in colonisation of the bile, which aren’t found in other *Campylobacter* species, *C. jejuni* and *C. coli.* Movement of *C. hepaticus* from the gastrointestinal tract, where it is most abundant, to the liver, and ultimately to the bile via the gall bladder could be an essential characteristic of disease. A better understanding of the role the *N-*glycan has on protein functions related to these survival and potential virulence mechanisms is needed. Extensive screening methods such as those used here alongside mutagenesis studies will be paramount in achieving this. This in turn will greatly aid the development of intervention methods to eliminate the economic burden associated with egg production losses linked to SLD while simultaneously improving animal welfare.

## Methods

### Bacterial cultivation

*Campylobacter hepaticus* HV10^T^ (NCTC 13823) isolated from a SLD-affected flock in Australia, *Campylobacter jejuni* 354, *Campylobacter coli* 54/2, *Campylobacter fetus* 54/3, *Campylobacter lari* 54/6, *Campylobacter upsaliensis* 54/7 were grown on Brucella agar (Amyl Media) with 5% horse blood (HBA) plates and incubated at 37 °C under microaerophilic conditions (85% N2, 10% CO2 and 5% O2) using CampyGen gas packs (Oxoid) for 48 h. All *Campylobacter* strains but *C. hepaticus* HV10^T^ are human clinical isolates obtained from RMIT University’s collection. Plates were harvested by flooding with ice-cold phosphate buffered saline (PBS) and gently resuspending cells using a spreader bar. *Escherichia coli* JM109 and *Salmonella* Typhimurium STM1^48^ were grown in Luria broth (tryptone (1%) (Sigma), yeast extract (0.05%) (Oxoid), and sodium chloride (1%). Cell suspensions were spun down at 8000 *g* for 5 min and the supernatant removed, cell pellets were washed twice with ice-cold PBS and stored at − 20 °C.

### *C. hepaticus* genome analysis

To determine *pgl* locus conservation between *C. hepaticus* HV10^T^*, C. jejuni* NCTC 11351*,* and *C. coli* RM2228, the *C. jejuni pglB* gene was used as query in BLAST searches. DNA sequences (genes upstream and downstream) surrounding *pglB* in each strain were uploaded to SnapGene (Dotmatics) and the amino acid sequences of ORF’s surrounding the *pglB* gene were determined by blastX. Amino acid sequences were used as queries in BLAST-UniProt to determine levels of amino acid sequence identity with well characterised *C. jejuni* subsp. *jejuni* NCTC11168.

### Total protein extraction for in solution digestion

Freshly grown C. *hepaticus* HV10^T^ cells were flooded with 2 mL PBS and harvested by scraping. Cells were washed three times in PBS then collected by centrifugation at 8000 *g* for 10 min and resuspended in 1 mL of ice-cold PBS. Cell suspensions were kept on ice and cells were lysed by sonication (Branson Digital Sonifier, 250 V) using six rounds sonication for 15 s with 30 s on ice between rounds. Samples were centrifuged at 8000 *g* for 10 min and supernatant was collected. The protein content of the lysed cell supernatant was determined using a Qubit™ Protein Assay kit: Q33211 (Invitrogen).

### Whole cell lysates for Western blotting

Freshly grown cells were harvested and washed 3 times in PBS. Samples were then collected by centrifugation at 8000 *g* for 5 min and cell pellets resuspended in 200 µL of PBS, vortexed and heated at 95 °C for 5 min. Samples were centrifuged at 8000 *g* for 5 min to remove the insoluble fraction and supernatants collected. Protein quantification was determined using a Qubit™ Protein Assay kit: Q33211 (Invitrogen). Protein samples were stored at − 20 °C.

### Western blotting

Whole cell lysates containing 25 µg or 15 μg of protein were suspended in 4 × Laemmli sample buffer (BioRad) and heated for 5 min at 95 °C. Samples were digested with 50 µg/mL proteinase K (Promega) for 1 h at 55 °C, loaded onto 8–16% precast polyacrylamide gels and separated at 100 V for 85 min. Proteins were transferred from SDS-PAGE gels to polyvinylidene difluoride (PVDF) membrane using an iBlot Dry Blotting system (Invitrogen). PVDF membranes were blocked at room temperature in 5% bovine serum albumin. Membranes were either probed with 10 μg/μL biotinylated soybean agglutinin (SBA) (Vector Laboratories) or with pooled (1:200) SLD positive and SLD negative sera samples in 0.1% PBS-T (1 × PBS, 0.1% Tween-20) containing 1% BSA. Positive sera samples were from five birds three-times vaccinated with *C. hepaticus* bacterin in a project approved by the Wildlife and Small Institutions Animal Ethics Committee of the Victorian Department of Economic Development, Jobs, Transport and Resources (approval number 14.16). SLD negative sera samples were from 5 Specific Pathogen Free chickens. Membranes were incubated for 1 h at RT. Membranes were then washed 3 times for 5 min in 0.1% PBS-T and transferred to secondary solutions either containing either streptavidin-HRP (invitrogen) in 0.1% PBS-T containing 1%BSA (Fig. 2), IRDye 800CW-streptavidin [1:5000] (LI-COR) in 0.1% PBS-T containing 1% SDS (Fig. 3), or anti-chicken IgY-HRP [1:5000] in 0.1% PBS-T containing 1% BSA (Fig. 3) and incubated for 1 h at RT. Membranes were washed 3 × 5 min in 0.1% PBST and rinsed for 5 min in 1 × PBS and signals detected using a ChemiDoc imaging system (Bio-Rad).

### Protein sample preparation for in-solution digestion

Extracted protein samples were prepared as previously described^34^. Briefly, total protein extracts as outlined above were acetone precipitated with 80% v/v ice-cold acetone from four separate *C. hepaticus* HV10^T^^6^ cultures overnight at − 20 °C. Protein precipitates were collected at 4000 *g* for 10 min (0 °C) and the resulting protein pellets resuspended in water to be precipitated with an additional round of acetone precipitation (80% v/v) at − 20 °C for 5 h. Twice precipitated protein pellets were collected at 6000 *g*, 15 min (0 °C) and then air dried.

### Trypsin digestion

Samples were resuspended in 100 µL of 5% sodium dodecyl sulfate (SDS) by boiling for 10 min at 95 °C. Samples were quantified by bicinchoninic Acid (BCA) assay and 100 µg of samples was reduced/alkylated sequentially with dithiothreitol (DTT) 10 mM followed by chloroacetamide (CAA) 40 mM. Reduced/alkylated samples were then prepared for digestion using Micro S-traps (ProtiFi) according to the manufacturer’s instructions. Samples were digested for 4 h with 3.33 mg of trypsin/lys-c mix (1:33 protease/protein ratio) and collected according to the manufacturer’s instructions. Samples were dried down and further cleaned up with homemade C18 Stage.

Tips^49^. Eluted peptides were divided into two aliquots, (1) 10 mg of C18 cleaned up samples used for input analysis and (2) the remaining 90 µg of peptides used for Zwitterionic Hydrophilic Interaction Liquid Chromatography (ZIC-HILIC) enrichment.

### *C. hepaticus* HV10^T^ Glycopeptide enrichment by ZIC-HILIC

Samples for ZIC-HILIC glycopeptide enrichment were resuspended in 80% acetonitrile, 1% TFA and glycopeptides enriched using homemade ZIC-HILIC StageTips as previously described ^50^. Briefly, ZIC-HILIC columns were first conditioned with 80% acetonitrile, 1% TFA and then samples were loaded onto columns before being washed with 80% acetonitrile, 1% TFA and glycopeptides eluted with Milli-Q water. Samples were dried and stored at – 20 °C until undergoing Liquid Chromatography Mass Spectrometry (LC–MS).

### LC–MS analysis of samples

Input control samples and ZIC-HILIC enriched samples were re-suspended in Buffer A* 0.1% trifluoroacetic acid, 2% acetonitrile and separated using a two-column chromatography set up composed of a PepMap100 C18 20 mm × 75 μm trap and a PepMap C18 500 mm × 75 μm analytical column (Thermo Fisher Scientific). Columns were coupled to either a Orbitrap Fusion™ Lumos™ Tribrid™ Mass Spectrometer equipped with a FAIMS Pro interface (Thermo Fisher Scientific) or a Exploris™ 480 Mass Spectrometer (Thermo Fisher Scientific). Input controls were analyzed on the Exploris 480 using 125-min gradients and the Fusion Lumos Tribrid using 145-min gradients while ZIC-HILIC enriched samples were analyzed only on the Fusion Lumos Tribrid using 145-min gradients. Separation gradients were run for each sample altering the buffer composition from 2% Buffer B (0.1% formic acid, 77.9% acetonitrile, and 2% DMSO) to 28% B over 106 or 126 min depending on the run length, then from 28 to 40% B over 9 min, then from 40 to 80% B over 3 min, the composition was held at 80% B for 2 min, and then dropped to 2% B over 2 min and held at 2% B for another 3 min. The Exploris™ Mass Spectrometer was operated in a data-dependent mode with a single Orbitrap MS scan (400–2000 m/z, and a resolution of 120 k) acquired every 3 s followed by MS/MS high energy collision dissociation (HCD) scans of precursors (stepped normalized collisional energy NCE 28%, 34% and 45%; maximal injection time of 100 ms, an Automatic Gain Control (AGC) set to a maximum of 1.5 × 10^5^ ions and a resolution of 30 k). The Lumos™ Mass Spectrometer was operated in a stepped Field Asymmetric Ion Mobility Spectrometry (FAIMS) data-dependent mode at three different FAIMS compensation voltages (CVs) − 25, − 45 and − 65 as previously described^34^. For each FAIMS CV a single Orbitrap MS scan (350–2000 m/z, maximal injection time of 50 ms, an AGC of maximum of 4 × 10^5^ ions and a resolution of 60 k) was acquired every 1.5 s followed by Orbitrap MS/MS HCD scans of precursors (NCE 30%, maximal injection time of 80 ms, an AGC set to a maximum of 1.25 × 10^5^ ions and a resolution of 30 k). HCD scans containing the oxonium ions (204.0867; 138.0545 and 366.1396 m/z) triggered three additional product-dependent MS/MS scans of potential glycopeptides; a Orbitrap electron-transfer/higher-energy collision dissociation EThcD scan (NCE 15%, maximal injection time of 250 ms, AGC set to a maximum of 2 × 10^5^ ions with a resolution of 30 k and using the extended mass range setting to improve the detection of high mass glycopeptide fragment ions^51^; a ion trap collision-induced dissociation (CID) scan (NCE 35%, maximal injection time of 40 ms, an AGC set to a maximum of 5 × 10^4^ ions) and a stepped collision energy HCD scan (using NCE 35%, maximal injection time of 250 ms, an AGC set to a maximum of 2 × 10^5^ ions and a resolution of 30 k). Data files were searched with MSfragger (v15)^52^, using the *C. hepaticus* HV10^T^ (uniparc: UP000093205) proteome. Open database searches were performed on input controls with the resulting “psm.tsv” files combined within R and only assignments with a MSfragger Hyperscores > 15 retained for delta mass visualization using ggplot2^53^. For Glycopeptide analysis ZIC-HILIC enriched samples were searched. Glycopeptide analysis of ZIC-HILIC enriched samples was accomplished by undertaking HCD and EThcD searches separately and combining the resulting “psm.tsv” files using R retaining only the best identification for each scan event and glycopeptides with a MSfragger Hyperscore > 15. Intensity- based absolute quantification (iBAQ) of input data sets was undertaken using MaxQuant v1.6.3.4 as described in^54^, searching against the reference *C. hepaticus* HV10 strain. The resulting search results, raw Data files and scripts are available via ProteomeXchange with identifier PXD028607.

### Database searching to characterise glycopeptides and their associated proteins

*C. hepaticus* peptides modified with the *N-*glycan were identified by database searching using NCBI BLAST and Uniprot peptide search. The number of glycosylation sites for each protein based on peptide matches and *N*-glycan sites were manually assigned according to the consensus sequon **D/E-X-N-X-S/T**. Predicted glycopeptide localisations were determined using PSORTb (vers. 3.0.3)^55^ and Gneg-mPloc^56^.

### Supplementary Information

Supplementary Information 1.

Supplementary Information 2.

## Data Availability

The proteomics datasets generated and analysed during the current study are available in the ProteomeXchange repository via identifier PXD028607. http://proteomecentral.proteomexchange.org/cgi/GetDataset?ID=PXD028607.
