# Author Correction: Characterisation of *N*-linked protein glycosylation in the bacterial pathogen *Campylobacter hepaticus*

**DOI:** 10.1038/s41598-024-59808-8

**Published:** 2024-04-25

**Authors:** Jamieson B. McDonald, Nichollas E. Scott, Greg J. Underwood, Daniel M. Andrews, Thi Thu Hao Van, Robert J. Moore

**Affiliations:** 1 School of Science, RMIT University, Bundoora West Campus, Bundoora, VIC Australia; 2 Department of Microbiology and Immunology, University of Melbourne at the Peter Doherty Institute for Infection and Immunity, Parkville, VIC Australia; 3 Bioproperties Pty Ltd, RMIT University, Bundoora West Campus, Bundoora, VIC Australia

Correction to: *Scientific Reports* 10.1038/s41598-022-26532-0, published online 05 January 2023

The original version of this Article contained an error in the legend of Figure 2.

“(A) SDS-PAGE of *C. hepaticus* HV10T, *C. jejuni* 354, *C. coli* 52/2 and *C. upsaliensis* 54/7 whole cell lysates (WCL) containing 25 µg of protein. (B) SBA lectin blot binding profiles to *N-*glycans present in WCL of *C. hepaticus* HV10T, *C. jejuni* 354, *C. coli* 52/2, *C. upsaliensis* 54/7 containing 25 µg of protein. (C) SDS-PAGE of *C. lari* 54/6, *C. fetus* 54/3 alongside negative controls, *S.* Typhimurium PT44. The equivalent amount of protein from WCL of the different *Campylobacter* species were also digested with proteinase K (P.K).Whole cell lysates were separated by 8–16% SDS-PAGE and either developed with SimplyBlue™ SafeStain or transferred to PVDF membranes for blotting. (D) SBA lectin blot binding profiles to N-glycans present in WCL of *C. lari* 54/6, *C. fetus* 54/3, *C. coli* 52/2, *E. coli* JM109 and *S.* Typhimurium PT44 containing 25 µg of protein.”

now reads:

“(A) SDS-PAGE of *C. hepaticus* HV10T, *C. jejuni* 354, *C. coli* 52/2 and *C. upsaliensis* 54/7 whole cell lysates (WCL) containing 25 µg of protein. (B) SBA lectin blot binding profiles to *N-*glycans present in WCL of *C. hepaticus* HV10T, *C. jejuni* 354, *C. coli* 52/2, *C. upsaliensis* 54/7 containing 25 µg of protein. (C) SDS-PAGE of *C. lari* 54/6, *C. fetus* 54/3 alongside negative control, *S.* Typhimurium PT44. The equivalent amount of protein from WCL of the different *Campylobacter* species were also digested with proteinase K (P.K).Whole cell lysates were separated by 8–16% SDS-PAGE and either developed with SimplyBlue™ SafeStain or transferred to PVDF membranes for blotting. (D) SBA lectin blot binding profiles to N-glycans present in WCL of *C. lari* 54/6, *C. fetus* 54/3 and *S.* Typhimurium PT44 containing 25 µg of protein.”

Additionally, the original version of this Article contained errors in the legend of Figure 5.

“Ion-trap CID-MS spectra of two *C. hepaticus* HV10T glycopeptides. Spectra represent fragmented ions produced from the *N-*glycan. (A) *C. hepaticus* HV10T glycopeptide 23IQGTIAQIYDNNK36 with a delta mass of 1405.56 Da. (B) *C. hepaticus* HV10T glycopeptide 266AALAEGEANATIISAK282 with a delta mass of 1405.56 Da. Above is the heptasaccharide structure linked to the peptide drawn using the symbol nomenclature for glycans SNFG. The MS/MS spectra display fragmented ions generated from the glycan with the structure HexNAc-HexHAc-[Hex]-HexNAc-HexHAc-HexNAc-diNAcBac.”

now reads:

“Ion-trap CID-MS spectra of two *C. hepaticus* HV10^T^ glycopeptides. Spectra represent fragmented ions produced from the N-glycan. (A) *C. hepaticus* HV10^T^ glycopeptide ^266^AALAEGEANATIISAK^282^ with a delta mass of 1405.56 Da. (B) *C. hepaticus* HV10^T^ glycopeptide ^23^IQGTIAQIYDNNK^36^ with a delta mass of 1405.56 Da. Above is the heptasaccharide structure linked to the peptide drawn using the symbol nomenclature for glycans SNFG. The MS/MS spectra display fragmented ions generated from the glycan with the structure HexNAc-HexHAc-[Hex]-HexNAc-HexHAc-HexNAc-diNAcBac.”

The original Article has been corrected.

