# Supplementary material for: Characterisation of N-linked protein glycosylation in the bacterial pathogen Campylobacter hepaticus
Source: Sci Rep. 2023 Jan 5;13:227. doi: 10.1038/s41598-022-26532-0 (PMC9816155; doi:10.1038/s41598-022-26532-0)
Supplement: Supplementary file 1 — Supplementary Information 1. [file 41598_2022_26532_MOESM1_ESM.pdf]

**Supplementary Information**

**Table of contents**

| Supplementary data | Description |
| --- | --- |
| Supplementary Figure 1 | Uncropped version of Figure 2. The well and bottom of gels/blots are visible. |
| Supplementary Figure 2 | Uncropped version of Figure 3. The well and bottom of gels/blots are visible. |
| Supplementary Figure 3 | Open searching shows additional modification, zoomed in shows evidence of formylated glycan (corresponding to the delta mass 1433). |
| Supplementary Figure 4 | iBAQ plot of *C. hepaticus* HV10$^T$ glycoproteins |
| Supplementary Table 1 | Combined MSfragger searches of *C. hepaticus* HV10$^T$ HILIC enriched glycopeptides (tab 1) and unenriched glycopeptides (tab2) with stepped FAIMS |
| Supplementary Table 2 | List of *C. hepaticus* HV10$^T$ *N*-glycoproteins and their predicted localisation and abundance. |

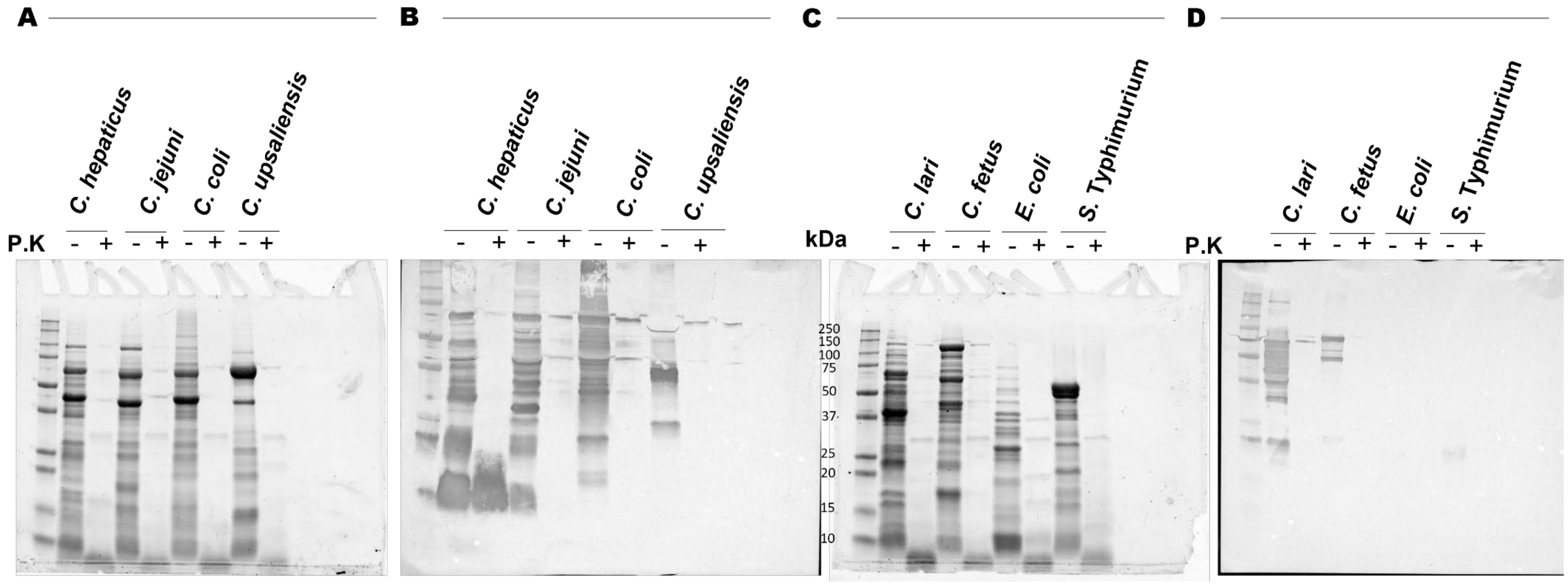


**Supplementary Figure 1. (A)** SDS-PAGE of *C. hepaticus* HV10^T^, *C. jejuni* 354, *C. coli* 52/2 and *C. upsaliensis* 54/7 whole cell lysates (WCL) containing 25 µg of protein. **(B)** SBA lectin blot binding profiles to *N*-glycans present in WCL of *C. hepaticus* HV10^T^, *C. jejuni* 354, *C. coli* 52/2, *C. upsaliensis* 54/7 containing 25 µg of protein. **(C)** SDS-PAGE of *C. lari* 54/6, *C. fetus* 54/3 alongside negative controls, *E. coli* JM109 and *S.* Typhimurium PT44 . **(D)** SBA lectin blot binding profiles to *N*-glycans present in WCL of *C. lari* 54/6, *C. fetus* 54/3, *C. coli* 52/2, *E. coli* JM109 and *S.* Typhimurium PT44 containing 25 µg of protein. The equivalent amount of protein from WCL of the different *Campylobacter* species were also digested with proteinase K (P.K).Whole cell lysates were separated by 8-16% SDS-PAGE and either developed with SimplyBlue™ SafeStain or transferred to PVDF membranes for blotting.

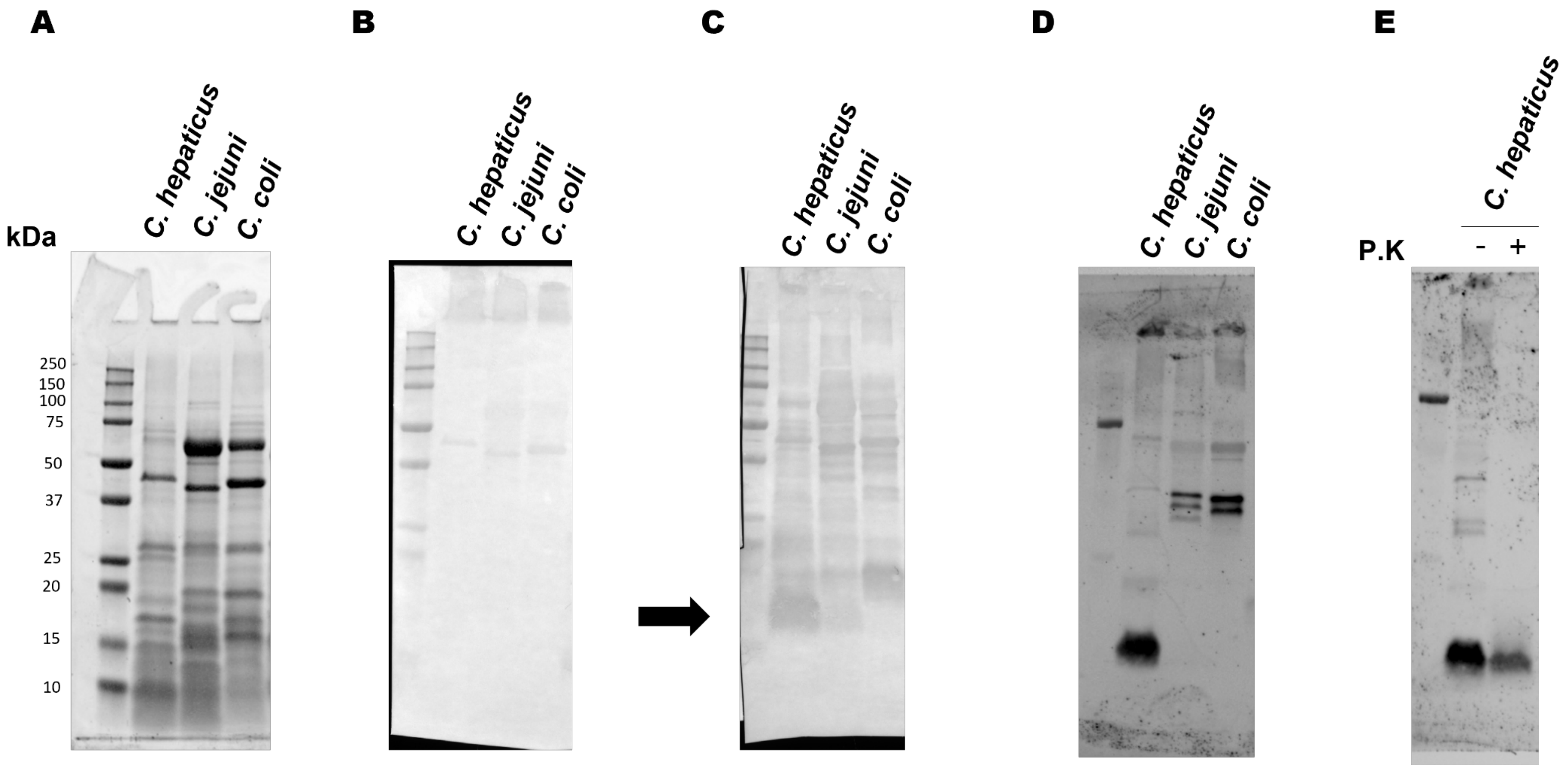


**Supplementary Figure 2.** Binding of serum IgY antibodies from *C. hepaticus* HV10$^T$ bacterin vaccinated birds to *Campylobacter* whole cell lysates. Wells were loaded with whole cell lysates containing 15 µg of protein, **(A)** SDS-PAGE loading control, **(B)** immunoblot with *C. hepaticus* negative sera (1:200) **(C)** Immunoblot of *C. hepaticus* HV10$^T$ positive (3 times vaccinated with *C. hepaticus* HV10$^T$ bacterin) sera (1:200). The arrow points to an immunodominant band corresponding to the ≈15 kDa non-proteinaceous signal suspected to be *C. hepaticus* HV10$^T$ LOS **(D)** SBA lectin blot **(E)** SBA lectin blot after proteinase K digestion (P.K).

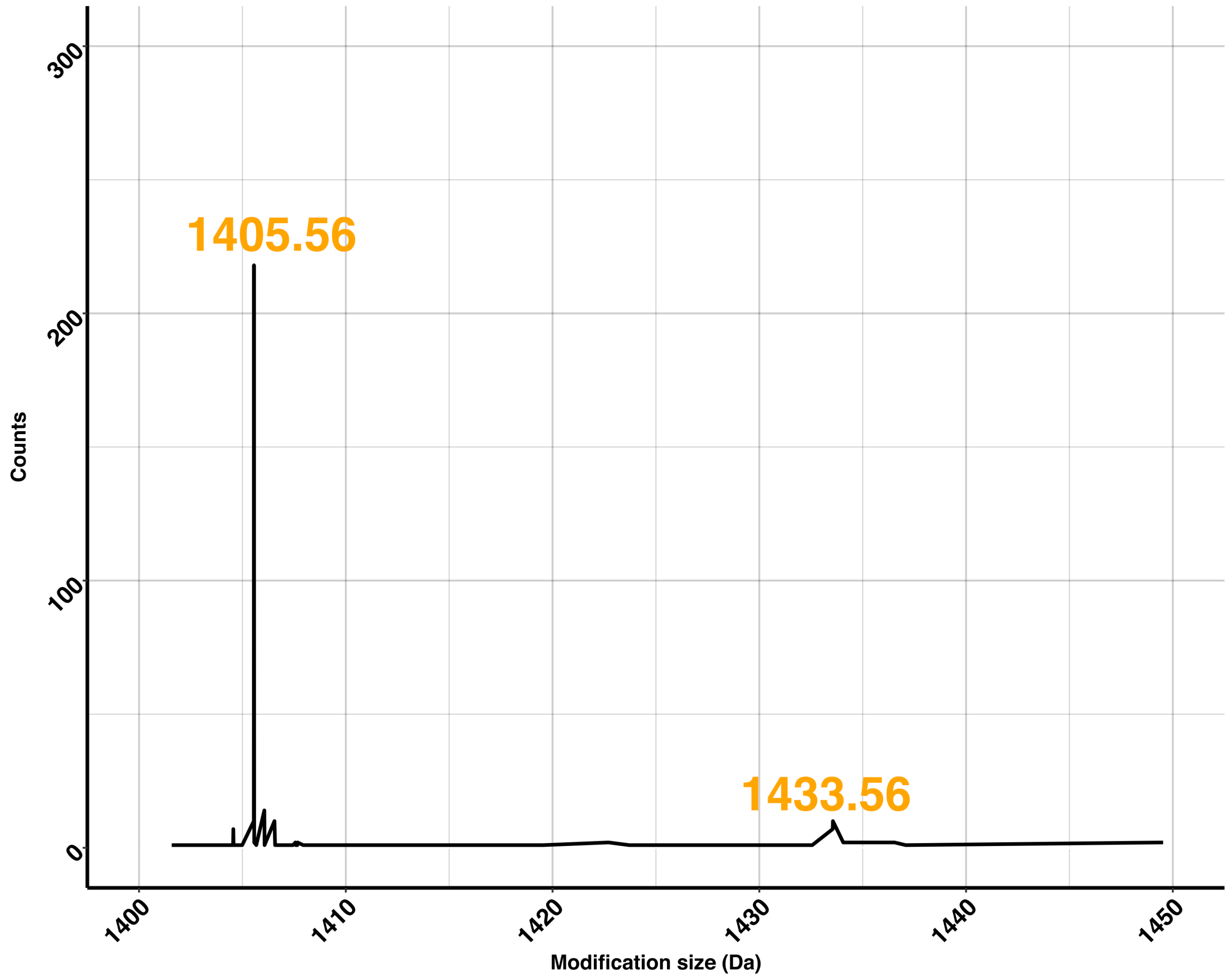


**Supplementary Figure 3.** Open searching shows additional modification, zoomed in shows evidence of formylated glycan (corresponding to the delta mass 1433).

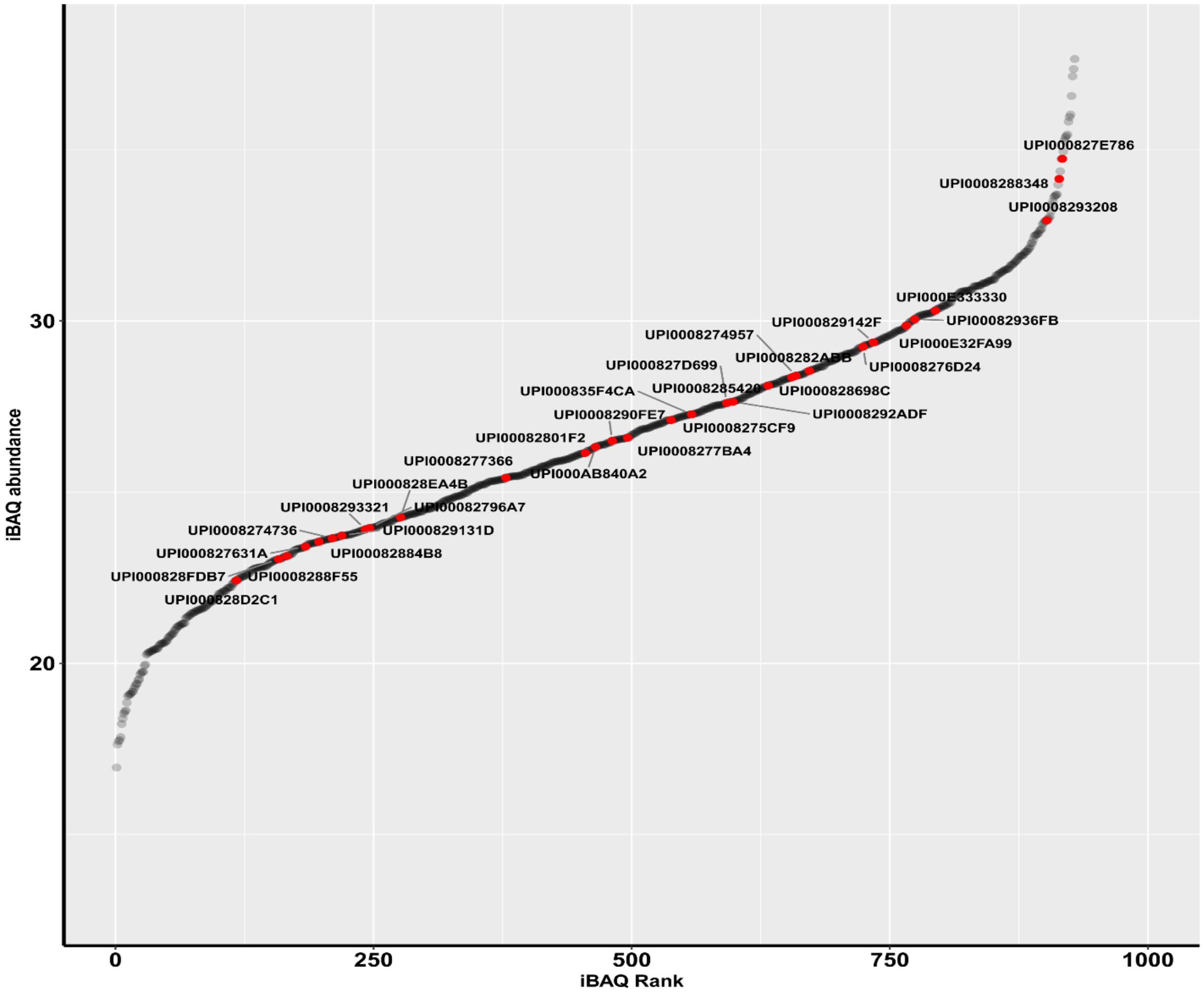


Supplementary Figure 4. iBAQ plot of *C. hepaticus* HV10$^{T}$ glycoproteins

**Supplementary Table 1**. Combined MSfragger searches of *C. hepaticus* HV10$^{T}$ HILIC enriched glycopeptides (tab 1) and unenriched glycopeptides (tab2) with stepped FAIMS. See the accompanying Excel file.

Supplementary Table 2. List of *C. hepaticus N*-glycoproteins and their predicted localisation and abundance.

| **Protein Name (UniProt accession)** | **Similarity to *C. jejuni* (UP000411585) proteins** | **Glycosylation sites** | **Peptide sequence/N-glycan sites** | **Localisation** | **Abundance in sample (most (1) abundant to least (29))** |
|---|---|---|---|---|---|
| **A0A6I1PJI0** Mechanosensitive ion channel family protein | 84.9% identity - **Q0PBR6** Mechanosensitive ion channel family protein | 1 | $^{52}$NEDQ**N**SSTFKG$^{62}$ | Inner Membrane | 26 |
| **A0A424Z1X3** Efflux RND transporter periplasmic adaptor subunit | 76.8%% identity **Q0P9M0** multidrug efflux transporter periplasmic adapter subunit CmeE | 3 | $^{267}$AIFEN**N**NSSLLPGAFATITSDAFIQKN$^{293}$<br>$^{197}$IDQ**N**QTQYKG$^{206}$<br>$^{98}$ANVDIAYGQTLMAQANFENASKDF**N**RS$^{124}$ | Inner membrane | 24 |
| **A0A6A7JQB7** Rod shape-determining protein MreC | 89.6% identity - **Q0PBM8** Rod shape-determining protein | 1 | $^{87}$ILEDQ**N**STKY$^{96}$ | Inner membrane | 28 |
| **A0A424Z0L7** MotE family protein | 82.0% identity – **Q0P8C1**Putative periplasmic protein | 2 | $^{164}$NLDN**N**LSN$^{172}$<br>$^{68}$EAEV**N**ATLAK$^{l78}$ | Inner membrane | 9 |
| **A0A6A7JTI1** OmpA family protein | 89.6% identity - **Q0PAR8** OmpA family protein | 3 | $^{166}$LDD**N**ITVDEK$^{176}$<br>$^{108}$DL**N**STLDDKDKQ$^{119}$<br>$^{93}$AELEA**N**ITNYKQ$^{104}$ | Outer membrane | 27 |

| | | | | | |
|---|---|---|---|---|---|
| **A0A6A7JUB4** AMIN domain-containing protein | 84% identity - **Q0P7V9** AMIN domain-containing protein | 3 | $^{67}$VDLDI**N**KS$^{74}$<br>$^{24}$MDE**N**ISLAILPQFQKE$^{39}$<br>$^{94}$VLDVSVTIPENGSKQES**N**ITANVEIPLEVSKI$^{125}$ | Inner membrane | 19 |
| **A0A6A7JRW2** Peptidylprolyl isomerase | 82.1% identity - **Q0PAI5** putative periplasmic protein | 2 | $^{124}$EFQNINGDF**N**KT$^{135}$<br>$^{305}$DL**N**ISESDISYPIDLLNKA$^{323}$ | Inner membrane | 15 |
| **A0A424Z177** DUF459 domain-containing protein | 77.8% identity- **Q0PAQ7** Putative periplasmic protein | 1 | $^{325}$FKEE**N**ASK$^{333}$ | Inner Membrane | - |
| **A0A6A7JSF1** M23 family metallopeptidase | 83.4% identity - **Q0PC06** Putative peptidase M23 family protein | 1 | $^{70}$DD**N**NTMVIADEKI$^{82}$ | Outer membrane | 18 |
| **A0A424Z2F4** Nitrate reductase, electron transfer subunit | 82.7% identity - **Q0PAA7** Nitrate reductase cytochrome c-type subunit | 1 | $^{40}$LVEA**N**FTSLQPGESTRL$^{56}$ | Periplasm | 7 |
| **A0A424Z3D1** Prohibitin family protein | 96.7% identity - **Q0PBN7** Putative transmembrane protein | 1 | $^{266}$AALAEGEA**N**ATIISAKG$^{282}$ | Inner membrane | 11 |
| **A0A6A7JR14** MetQ/NlpA family ABC transporter substrate-binding protein | 85.6% identity – **Q0PAB8** Putative NLPA family lipoprotein. | 1 | $^{54}$EFTDYVLPNLAVDNAEIDANFFQHTPYLEEF**N**KS$^{87}$ | Inner Membrane | 1 |

| | | | | | |
|---|---|---|---|---|---|
| | Glycosylation unique to *C. hepaticus* or not yet identified in *C. jejuni* | | | | |
| A0A6A7JRD9 Lytic transglycosylase SLT domain-containing protein | 84.5% identity - Q0PA47 Putative secreted transglycosylase | 2 | $^{326}$DANASELDALAKE$^{338}$ $^{93}$TILDANLTCQSIRL$^{106}$ | Periplasm | 16 |
| A0A424Z347 Efflux transporter outer membrane subunit (TolC family protein) | 80.5% identity - Q0PBE5 multidrug efflux transporter outer membrane channel CmeC | 1 | $^{38}$LGALDWEKENNNTINKE$^{54}$ | Outer membrane | 9 |
| A0A6A7JU28 Cytochrome c biogenesis protein CcsA | 85.5% identity - Q0P9N9 Putative cytochrome C biogenesis protein | 1 | $^{227}$IDENLTLSSSENLHFLNMLDGSNLKL$^{252}$ | Inner membrane | 29 |
| A0A6A7JRY0 Flagellar protein | Q0P863 81.2% Identity – Paralysed Flagellum protein | 2 | $^{494}$EGNFSAVLAYKD$^{505}$ $^{455}$DNNASFLHERY$^{465}$ | Cytoplasmic | 21 |
| A0A424Z0D4 SH3 domain containing protein | 72.8% identity - Q0P9V3 Putative lipoprotein Glycosylation unique to *C. hepaticus* or not yet identified in *C. jejuni* | 2 | $^{39}$LEFEQNVSILPKL$^{51}$ $^{89}$FDFNISVEKN$^{98}$ | Inner membrane | 22 |

| | | | | | |
|---|---|---|---|---|---|
| A0A424Z338 Tetratricopeptide repeat protein | 87.1% identity – Q0PBC0 Putative transmembrane protein | 1 | $^{167}$DF**N**QSLAALSLLDIKD$^{176}$ | Inner membrane | 25 |
| A0A6A7JQY3 ArsS family sensor histidine kinase/ HAMP domain containing family sensor histidine kinase | 78.2% identity Q0P8Z4 Two component sensor Glycosylation unique to *C. hepaticus* | 1 | $^{48}$NLSLFYEN**N**ISNAKI$^{62}$ | inner membrane | - |
| A0A6I1PIY8 PDZ domain-containing protein | 91.4% identity - Q0PB04 Putative secreted protease | 1 | $^{55}$TLAIVEQYYVEDQ**N**ISELVDKS$^{76}$ | Inner membrane | 4 |
| A0A424Z1P2 PDZ domain-containing protein | 83.5% identity - Q0P7Y5 putative periplasmic protein containing protein. Glycosylation unique to *C. hepaticus* or not yet identified in *C. jejuni* | 1 | $^{233}$DNQDL**N**ISTEIFAKD$^{247}$ | Inner membrane | 10 |
| A0A6A7JR07 Fibronectin type III domain-containing protein/ Ferrous iron transporter A | 80.5% identity Q0P8X7 Putative fibronectin domain containing protein. Glycosylation Unique to *C. hepaticus* or not yet identified in *C. jejuni* | 1 | $^{126}$IIEA**N**TTSRL$^{35}$ | Inner membrane | 6 |

| | | | | | |
|---|---|---|---|---|---|
| A0A6A7JSL4 Hypothetical protein | 76.5.31% identity – Q0PBT9 Putative periplasmic protein | 1 | [23]IQGTIAQIYDN**N**KT[36] | Unknown | 3 |
| A0A424Z2X7 Hypothetical protein | 84.3% identity - Q0PBE6 hypothetical protein Unknown | 1 | [18]EQFYSYFDQ**N**ISK[30] | Inner membrane | 2 |
| A0A6A7JUB7 Hypothetical protein | 72.3% identity - Q0PC48 putative lipoprotein | 1 | [66]NCGDF**N**RS[73] | Inner membrane | 29 |
| A0A424Z1B0 Hypothetical protein | 82.5% identity - Q0PAM0 Putative membrane protein | 1 | [46]AYES**N**TSVVKA[56] | Inner membrane | - |
| A0A424Z079 Hypothetical protein | 59.4% identity - Q0P9V5 putative periplasmic protein | 1 | [147]TQDLEDL**N**ASKS[158] | Unknown | 14 |
| A0A424Z1G3 Hypothetical protein | 75.2% identity - Q0P807 putative periplasmic protein | 1 | [192]TYSLDL**N**KT[200] | Unknown | 13 |
| A0A6I1PLU1 Hypothetical protein | 76% identity - Q0PAT0 putative integral membrane protein | 1 | [281]DN**N**FSVIQKE[290] | Inner membrane | - |

| | | | | | |
|---|---|---|---|---|---|
| A0A6A7JR47 Hypothetical protein | 60.8% identity – Q0PAB4 Putative periplasmic protein | 2 | $^{84}$DNDKQDN**N**ITIKD$^{96}$<br>$^{96}$DNKQNLENDN**N**ISTIKN$^{112}$ | Unknown | 17 |
| A0A424Z226 Hypothetical protein | 76.3% identity - Q0P9J9 Putative integral membrane protein | 1 | $^{95}$DN**N**QSFNSLIIDIKD$^{109}$ | Inner membrane | 23 |
| A0A6A7JQN8 Hypothetical protein | 75.5% identity - Q0PBB3 hypothetical protein | 1 | $^{90}$NINLYAIFLMTQNNSDL**N**HTIIQNK$^{114}$ | Unknown | 20 |
| A0A424Z0Y5 Hypothetical protein | 74.7% Identity - Q0PC23 Putative periplasmic protein | 2 | $^{167}$QDF**N**ASNNKD$^{176}$<br>$^{87}$LMQLEESNQNIEN**N**LSAEIQR$^{108}$ | Outer membrane | 5 |

# Protein identity was determined by NCBI BLAST and Uniprot peptide search; no. of glycosylation sites for each protein; the peptide sequences and *N*-glycan site (manually assigned) based on consensus sequon **D/E-X-N-X-S/T**. Localisation was determined using Gneg-mPloc & PSORTb (vers. 3.0.3). Glycoprotein abundance in analysed samples was determined using iBAQ plot and ranked from most (1) abundant to least (29) abundant.  -: the abundance of 4 glycoproteins could not be determined
